# Data on eye movements of glaucoma patients with asymmetrical visual field loss during free viewing

**DOI:** 10.1016/j.dib.2023.109184

**Published:** 2023-05-10

**Authors:** Peter Reddingius, Daniel S. Asfaw, Vera M. Mönter, Nicholas D. Smith, Pete R. Jones, David P. Crabb

**Affiliations:** Division of Optometry and Visual Science, School of Health and Psychological Sciences, City, University of London, London EC1V 0HB, UK

**Keywords:** Glaucoma, Visual field, Eye movements, Eye tracking

## Abstract

This paper describes data from Asfaw at al. [1], which examined the eye movements of glaucoma patients (n=15) with pronounced *asymmetrical* vision loss (visual field loss worse in one eye). This allows for within-subject comparisons between the better and worse eye, thereby controlling for the effects of individual differences between patients. All patients had a clinical diagnosis of open angle glaucoma (OAG). Participants were asked to look at images of nature monocularly (free viewing; fellow eye patched) while gaze was recorded at 1000 Hz using a remote eye tracker (EyeLink 1000). Raw and processed eye tracking data are provided. In addition, clinical (visual acuity, contrast sensitivity and visual field) and demographic information (age, sex) are provided.

**Specifications Table**

**Table:** 

Subject	Ophthalmology
Specific subject area	Detecting visual field loss in glaucoma patients (using eye tracking data)
Type of data	Table Image Raw data files (ASCII format files)
How the data were acquired	Eye movements of the better and worse eye (as defined by visual field status) were acquired monocularly using an EyeLink 1000 eye tracker. Visual acuity was measured with the early treatment diabetic retinopathy study chart and contrast sensitivity was measured with a Pelli-Robson chart. Visual field data was acquired using Humphrey field analyser (Carl Zeiss Meditec, CA, USA).
Data format	Raw Filtered
Description of data collection	Fifteen participants with asymmetrical glaucomatous visual field loss freely viewed 120 images with each eye monocularly. Participants were seated with their chin in a chinrest at 60 cm from the screen
Data source location	School of Health and Psychological Sciences at City, University of London, London, United Kingdom
Data accessibility	Repository name: Zenodo Data identification number: 10.5281/zenodo.7761477 Direct URL to data: https://zenodo.org/record/7761477
Related research article	Asfaw, D. S., Jones, P. R., Mönter, V. M., Smith, N. D., & Crabb, D. P. (2018). Does glaucoma alter eye movements when viewing images of natural scenes? A between-eye study. *Investigative Ophthalmology & Visual Science, 59*(8), 3189–3198. 10.1167/iovs.18-23779

**Value of the Data**

• Raw and processed eye tracking data from 15 participants with a median {interquartile range [IQR]} age of 66 {63, 79} years can be used by other researchers investigating eye movement differences between eyes with different stages of glaucomatous visual field loss.
• The data allows for within-subject comparisons between eyes with greater and lesser visual field defects, thereby controlling for any individual differences such as cognitive skills and health status.
• This information can be useful to develop new algorithms for the detection and/or monitoring of visual field deficits using eye movements.

## 1 Objective

Glaucomatous visual field loss has been shown to cause changes in the eye movements (versus age-similar controls) during a variety of everyday tasks that include: reading [2,3], face recognition [4], visual search [5], watching videos [6] or viewing images [7]. However, many of these studies exhibit great individual differences in eye movements that may obscure the effects of the visual field defect *per se*. In the present study, participants had asymmetrical visual field loss, allowing the better seeing eye (with the better visual field status) to serve as the (within-subject) control for the worse eye. These data can be used to compare the eye movements of eyes with higher levels of glaucomatous visual field loss and eyes with lower levels as a within subject design study.

## 2 Data Description

Eye movement data were collected to assess whether glaucomatous visual field defects affect eye movements [1]. Each participant passively viewed 540 still images of nature that were either in full-colour (N = 180) or greyscale (N = 360). These images were displayed for a random duration ranging from 3 to 5 seconds. The dataset contains raw gaze data and processed eye movement data. The dataset also includes the visual field, acuity, contrast sensitivity, sex and age of the participants.

### 2.1 Participants

Fifteen glaucomatous individuals with pronounced asymmetric (between-eye) differences in visual field loss were recruited from a database of volunteers. All participants had an established clinical diagnosis of open angle glaucoma: an age-related disease of the optic nerve that causes a progressive loss of visual function [8]. Participants were eligible for the study if they had a distinct between-eye asymmetry in their visual field loss. This was defined as meeting at least one of the following criteria:

• A between-eyes difference in mean deviation (MD, the mean difference in sensitivity from normally sighted age-matched control data) of at least 6 dB or more
• A between-eyes difference in glaucoma severity of at least one stage, as measured by the Glaucoma Staging System 2 (GSS2) [9]

Fourteen participants satisfied both criteria. One participant had an inter-eye MD difference of 4.7 dB, but a two stage GSS2 difference. A visual representation of this can be found in Fig. 1 of Asfaw at al. [1].

### 2.2 Clinical Vision Tests

Distance visual acuity was measured monocularly, using the Early Treatment Diabetic Retinopathy Study (ETDRS) chart. All participants had a visual acuity of 0.18 logMAR or better (except the left eye of participant 14). Contrast sensitivity was measured monocularly using the Pelli-Robson chart (Precision vision ltd, Woodstock IL, USA).

All participants performed static threshold perimetry, monocularly in each eye, using a Humphrey Field Analyser (HFA; Carl Zeiss Meditec, Dublin, CA, USA) running the SITA Standard algorithm with Goldmann III stimuli. Every participant performed *both* a 10-2 and a 24-2 grid in each eye. The MD values and the greyscales of the 24-2 visual field test are shown in Fig. 1 of Asfaw at al. [1] and were used to determine which eye was ‘better’ or ‘worse’. Pointwise sensitivity values for the 10-2 and 24-2 tests are each provided in separate files (“HFA <10-2/24-2> sensitivities.csv”). Also included are the corresponding greyscale images for each participant, as images. The greyscale image files also include the image for the integrated visual field (IVF) which was calculated from the two monocular visual fields using binocular summation [10], [11], [12]

Summary measures of the visual field tests (HFA MD, pattern standard deviation [PSD] and visual field index [VFI]) are included in a single comma-separated file (“Master data.csv”), along with the contrast sensitivity, visual acuity, and basic demographic information. A description of each of the fields in this file can be found in Table 1.

**Table 1:** Description of the fields in the "Master data.csv" file.

Field	Description
participant_id	The id the participant was assigned for the experiment (*P1 to P15*)
age	Age of the participant at time of testing (*years*)
sex	Sex of the participant (“*M*” *or* “*F*”)
VA_L and VA_R	Visual acuity of the participant for the left (L) and right (R) eye respectively (*logMAR*)
CS_L and CS_R	Contrast sensitivity of the participant for the left and right eye respectively (*logCS*)
better_eye	Which eye was better according to the MD score (“*L*” *or* “*R*”)
block1_eye	Which eye was tested first (“*L*” *or* “*R*”)
R_MD_242, R_PSD_242, R_VFI_242	The MD, PSD and VFI respectively of the right eye for the 24-2 test (*dB*)
L_MD_242, L_PSD_242, L_VFI_242	The MD, PSD and VFI respectively of the left eye for the 24-2 test (*dB*)
IVF_MD_242, IVF_PSD_242, IVF_VFI_242	The MD, PSD and VFI respectively of the integrated visual field (IVF) of the 24-2 test (*dB*)
R_MD_102, R_PSD_102	The MD and PSD respectively of the right eye for the 10-2 test (*dB*)
L_MD_102, L_PSD_102	The MD and PSD respectively of the left eye for the 10-2 test (*dB*)
IVF_MD_102, IVF_PSD_102	The MD and PSD respectively of the IVF for the 10-2 test (*dB*)

The Individual Differential Light Sensitivity (DLS) values, in decibels, for each of the 54 test points in the 24-2 and 68 test points of the 10-2 are included in two .csv files (“HFA 24-2 sensitivity values.csv” and “HFA 10-2 sensitivity values.csv”). The values are stored in a single row but can also be visualised in a visual field map as shown in Fig. 1 of Asfaw et al. [1]. The row names of the files indicate the participant id, including “L” or “R” for the left or right eye respectively. The column names indicate the sensitivity value read from top to bottom, left to right, starting with “s1” which is the top left sensitivity value to the bottom right value “s54” for the 24-2 or “s68” for the 10-2. Visualised examples of the visual field are included in the data as a single image file for each participant for each test. These maps can be used to relate the direction of eye movements to visual field defects.

### 2.3 Raw Gaze Data

Eye movements were measured using an EyeLink 1000 remote eye tracker (SR Research Ltd., Ontario, Canada). By default, the EyeLink 1000 outputs data to an .edf (EyeLink Data File) file format: a form of raw binary file. However, for convenience these files have been converted to .asc files using EDF2ASC: a program supplied by SR Research. These .asc files can be read in an ordinary text viewer, and contain the entire recorded eye location, pupil size and eye tracking events (fixations, saccades and blinks). Other eye tracking information, such as calibration and synchronization information are also stored in the .asc files. Each participant has their own individual .asc file. Minor edits have been made to the .asc files as the eye tracker was used for another experiment after this one, in order to prevent confusion, that other data were removed.

Detailed information about the .asc file format and structure are provided by the manufacturer (SR Research; https://www.sr-research.com).

### 2.4 Processed Eye Movement Data

The raw data contains the x and y gaze positions and estimated pupil diameter at each millisecond since starting the eye tracker. For convenience, additional files have also been provided, in which the raw data have also been processed to extract the key eye-movement events and image presentation times. These events include the starts and ends of fixations, and the starts and ends of saccades, each determined using the EyeLink's internal algorithms. Each fixation end, for example, gives the start and end time, the duration, the mean x and y position, and mean pupil size during the fixation. While each saccade end specifies the amplitude, velocity, duration, start and end time, and start and end location of the saccade. The extracted data contains 19 fields, a description of these fields can be found in Table 2. Not all fields contain data for every event, as some information is only presented for saccades or fixations. The processed eye movement files are stored in csv file format, with one file per participant (“<participant_id> - processed data.csv”).

**Table 2:** Description of the fields in the processed eye movement files of each participant.

Field	Included for:	Description
participant_id	All events	The participant id (*P1 to P15*)
eye	All events	The eye that was tested in the block (*“L” or “R”*)
block	All events	The study consisted of two blocks, each with 270 trial images.
trial	All events	The trial number (*1 to 270*)
event	All events	The event type (*fixation, saccade, start trial, end trial*) that the eye tracker recorded
greyscale	All events	Shows “TRUE” if the image displayed in the trial is in greyscale
includedInAnalysis	All events	Shows “TRUE” if this trial was analysed in the paper by Asfaw et al. [1]
startTime	All events	Start time of the event (*in ms since the* E*yeLink eye tracker software was started*)
endTime	All events, except start trial	End time of the event (*in ms since the* E*ye*L*ink eye tracker software was started*)
duration	All events, except start trial	The duration of the event (*in ms*)
meanX	Fixations only	The mean X position of the eye during the fixation (*1 to 1600 pixels*)
meanY	Fixations only	The mean Y position of the eye during the fixation *(1 to 1200 pixels*)
startX	Saccades only	The X position of the eye at the start of the event (*1 to 1600 pixels*)
startY	Saccades only	The Y position of the eye at the start of the event (*1 to 1200 pixels*)
endX	Saccades only	The X position of the eye at the end of the event (*1 to 1600 pixels*)
endY	Saccades only	The Y position of the eye at the end of the event (*1 to 1200 pixels*)
amplitude	Saccades only	Size of the saccade (*degrees visual angle*)
peakVelocity	Saccades only	Peak of gaze velocity during the saccade (*degrees visual angle per second*)
pupilArea	Fixations only	The average pupil size during the fixation (*arbitrary unit defined by EyeLink, range 100 to 100000*)

## 3 Experimental Design, Materials and Methods

### 3.1 Apparatus

The participant was seated with their head stabilised using a chin rest 60 cm away from the monitor. Images were presented on a 56 cm (diagonal) CRT computer monitor (Liyama Vision Master Pro 514; Liyama Corporation, Tokyo, Japan) running at 100 Hz with a resolution of 1600 by 1200 pixels. At the 60 cm distance from the screen, the size of the screen was 34 degrees visual angle horizontally, and 26.8 degrees visual angle vertically. Participants were appropriately refractively corrected, as required, using trial frames to make sure that any restrictions in the visual field due to spectacles were constant across participants. Eye movements were recorded using the EyeLink 1000 remote eye tracker (SR Research Ltd., Ottowa, ON, Canada) which records at 1000 Hz with a spatial precision of ≤ 0.5°. Before the first trial, gaze calibration was performed using a nine-point calibration grid and repeated until the software reported the calibration in validation to be ‘good’. Between each trial a drift check was introduced, which allowed for a recalibration if substantial drift was detected. The participants were asked to view the images freely as a slideshow. Participants only needed to use the keyboard to indicate that they wanted to continue to the next trial.

### 3.2 Stimuli

There were 540 unique images of nature that were either in full colour (180) or in greyscale (360). These images were extracted from a nature documentary (Planet Earth; BBC Television, London, UK) and showed outdoor natural scenes with animals and flowers, including underwater images. They were displayed full screen on the 1600 × 1200 pixel monitor.

Participants took part in two blocks, one block for each eye. During each block, the participant viewed 270 images. These 270 images contained 150 images that were unique to each block and 120 images that were shown in both blocks. The 120 images were shown to both eyes monocularly and were used to compare the pattern of eye movements in the better and worse eye in the paper by Asfaw et al. [1]. The 150 unique images were used to prevent the viewer from recognising the 120 images that were shown to both eyes. The images can be made available upon request to the corresponding author.

After each block the participants also looked at images with text on them (used in the paper by Smith et al. [3]) and performed several smooth pursuit trials. For clarity, these data have been taken out of the files.

## Ethics Statements

The Optometry Proportionate Review Committee at City, University of London approved the study. The research was carried out in accordance with the Declaration of Helsinki and all participants provided written informed consent.

## CRediT authorship contribution statement

**Peter Reddingius:** Data curation, Writing – original draft. **Daniel S. Asfaw:** Formal analysis, Visualization. **Vera M. Mönter:** Methodology, Investigation. **Nicholas D. Smith:** Methodology, Software. **Pete R. Jones:** Writing – review & editing, Supervision. **David P. Crabb:** Conceptualization, Methodology, Writing – review & editing, Supervision.

## Declaration of Competing Interest

The authors declare that they have no known competing financial interests or personal relationships that could have appeared to influence the work reported in this paper.

## Data Availability

Data on eye movements of glaucoma patients with asymmetrical visual field loss during free viewing. (Original data) (Zenodo).
